# Cancer Risk in Marfan Syndrome: A Swedish Population‐Based Cohort Study

**DOI:** 10.1002/ijc.70541

**Published:** 2026-05-13

**Authors:** Ida Nordgren, Anna Skarin Nordenvall, Alexandra Wachtmeister, Fulya Taylan, Yunxia Lu, Christina Norrby, Anna Lindstrand, Giedre Grigelioniene, Giorgio Tettamanti

**Affiliations:** ^1^ Department of Molecular Medicine and Surgery Karolinska Institutet Stockholm Sweden; ^2^ Department of Radiology Karolinska University Hospital Stockholm Sweden; ^3^ Department of Population Health and Disease Prevention & Department of Epidemiology and Biostatistics, Joe. C. Wen School of Population Health & Public Health University of California Irvine Irvine California USA; ^4^ Department of Clinical Genetics and Genomics, Karolinska University Laboratory Karolinska University Hospital Stockholm Sweden; ^5^ Unit of Epidemiology, Institute of Environmental Medicine Karolinska Institutet Stockholm Sweden

**Keywords:** adult cancer risk, cancer predisposition syndrome, childhood cancer risk, *FBN1*, Marfan syndrome

## Abstract

Marfan syndrome is an autosomal dominant connective tissue disorder caused by pathogenic variants in the fibrillin‐1‐encoding gene. The cancer risk in Marfan syndrome is not fully understood, but recent reports have suggested an increased risk in adults. This study assessed cancer risk in Marfan syndrome using population‐based data from the Swedish National registers.

We performed a register‐based nationwide matched cohort study of 1544 individuals with Marfan syndrome, born 1930–2017. Each individual was matched to 50 comparisons by birth year, sex and birth county. Cancer risk was estimated using Cox proportional hazard models, expressed as hazard ratios (HRs) with 95% confidence intervals (CIs). All cancer cases diagnosed before age 20 years were classified as childhood cancer, and those diagnosed at age 20 years or older were classified as adult cancer.

The overall risk of adult cancer was not increased in individuals with Marfan syndrome (HR 1.00, 95% CI 0.78–1.27). However, there was an increased risk of endocrine tumours in adults with Marfan syndrome (HR 2.86, 95% CI 1.40–5.85). Furthermore, an over two‐fold increased risk of cancer was observed among children with Marfan syndrome (HR 2.44, 95% CI 1.25–4.80).

In this study, we found an increased cancer risk in children with Marfan syndrome. In contrast to previous reports, we did not detect an increased overall cancer risk in adults with Marfan syndrome, but an elevated site‐specific risk of endocrine tumours. Further, larger studies are warranted to evaluate the lifetime cancer risk in Marfan syndrome at different ages.

## Abstract (highlights)

What's new?

The cancer risk in Marfan syndrome, an autosomal dominant connective tissue disorder, largely remains to be explored. In this population‐based matched cohort study of 1544 Swedish patients, the overall cancer risk in adults with Marfan syndrome showed no significant increase, except for the risk of endocrine tumours with a nearly threefold increase. Moreover, children with Marfan syndrome had a more than twofold higher risk of developing cancer, mainly driven by solid tumours. While the absolute risks of both childhood and adult cancer remained low, the findings provide novel insights into Marfan syndrome as a potential cancer predisposing syndrome.

## Graphical abstract

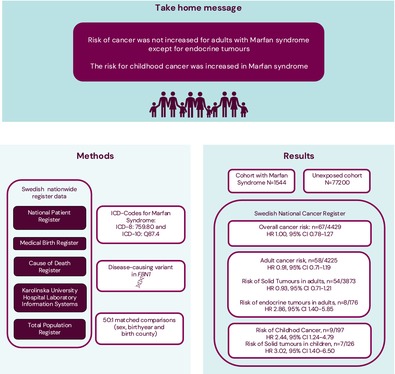

**List of abbreviations**
CIs: Confidence intervals
DOR: Cause of death register
HRs: Hazard ratios
ICD: International classification of diseases
MBR: Medical birth register
MFS: Marfan syndrome
NCR: National cancer register
NPR: National patient register
TPR: Total population register

## 1 Introduction

Marfan syndrome (MFS), *OMIM #154700* [1], is an autosomal dominant connective tissue disorder affecting multiple organ systems, including the skeleton, cardiovascular system, eyes, lungs, and skin. MFS is caused by pathogenic variants in the *FBN1* gene, located on chromosome 15q21.1 (*OMIM* *134797) [2]. *FBN1* encodes for Fibrillin‐1, a protein that polymerizes into microfibrils that are important for the integrity and function of connective tissue. These microfibrils control the bioavailability of TGF‐β signalling that is involved in several cellular processes, for example, proliferation, apoptosis, and differentiation [3, 4, 5].

MFS occurs in approximately 1:3000–1:5000 individuals [6, 7] and about 25% of *FBN1* pathogenic variants arise de novo [8]. There are more than 3000 different pathogenic variants described in *FBN1* [9], some of which are associated with a more severe phenotype [8, 10]. The clinical phenotype can vary, even within families, ranging from mild skeletal deformities to early death due to severe cardiovascular catastrophes [8].

Cardiovascular manifestations of MFS include dilation or dissection of the aortic root and valvopathies, particularly mitral valve prolapse [11]. Skeletal features include craniofacial characteristics, long and thin extremities, arachnodactyly, pectus carinatum or excavatum, and scoliosis [12]. Eye manifestations often occur at early ages and include lens dislocation, myopia, retinal detachment, and cataract development [13].

Life expectancy is reduced in MFS, mainly for males, due to the common and often severe cardiovascular manifestations. Aortic dilatation and its associated complications are the main causes of death. In recent years, life expectancy has increased due to improved outcomes of interventions, better follow‐up, and advances in medical therapy. Reported mean life expectancy in MFS increased from 32 years in 1972 to 63 years for men and 73 years for women in 2018 [14, 15].

With increasing survival, there is a growing need to evaluate the disease burden at older ages, including cancer risk during adulthood. Several case reports of individuals with MFS and cancer have been published: these include cancer types such as leukaemia, diffuse B‐cell lymphoma, myeloid sarcoma, non‐Hodgkin lymphoma, Wilms tumour, neuroblastoma, astrocytoma, testicular cancer, colon cancer, and osteosarcoma, among others [16, 17, 18, 19, 20, 21, 22]. In 2017, Hsu et al. conducted a nested case–control study in Taiwan of 93 individuals with MFS who developed cancer and reported a significantly increased risk of malignancies associated with MFS, with a highly elevated risk for head and neck tumours and urinary tract malignancies [23].

This study aimed to further investigate the risk of cancer in children and adults with MFS born in Sweden, using data from Swedish national registers and the Karolinska University Hospital's Clinical Genetics Laboratory Information System.

## 2 Materials and Methods

For studying the association between MFS and cancer, we used data from the Rare Diseases and Cancer Study (RADICA‐study), a large Swedish register‐based research study where several Swedish healthcare and demographic registers have been linked through the personal identification number (PIN) given to all Swedish residents [24].

### 2.1 Study Design and Participants

The study population comprised all individuals born in Sweden between January 1, 1930, and December 31, 2017, with a registered diagnosis of MFS in the National Patient Register (NPR), the Medical Birth Register (MBR), the Cause of Death Register (DOR), or the Karolinska University Hospital Laboratory Information Systems (Starlims). Individuals were identified using the International Classification of Diseases (ICD) codes 759.80 (ICD‐8) and Q87.4 (ICD‐10), or through a genetically confirmed pathogenic *FBN1* variant in the Starlims laboratory database.

The NPR has contained information on inpatient hospital visits since 1964, with nationwide coverage achieved by 1987. Outpatient visit data have been available since 2001 [25]. The MBR has collected data on birth characteristics, perinatal diagnoses, and congenital malformations since 1973 [26]. Information on cause and date of death has been recorded in the DOR since 1952. Since 1968, the total population register (TPR) has gathered information on dates of birth, death, emigration, birth county, and basic demographics data [27]. Genetic data from the Stockholm region, which accounts for approximately 23% of the Swedish population [28], have been stored in Starlims since 1992. In this study, data from Starlims covering the period 1992–2021 were used.

All individuals with pathogenic variants in *FBN1* were included in the study. Individuals with MFS who lacked a registered birth county were excluded. Every individual with MFS was matched to 50 comparisons by birth year, sex, and birth county, identified through TPR. All individuals were followed until the earliest occurrence of a first cancer diagnosis, emigration, death, or end of follow‐up (2017–12–31).

### 2.2 Identification of Cancer Cases

All individuals with MFS and their matched comparisons were linked to the National Cancer Register (NCR), which has collected nationwide data on cancer diagnoses since 1958. Reporting of malignant tumours, leukaemias, and selected premalignant and benign tumours to the NCR is mandatory for all healthcare providers [29]. Cancer cases were identified in NCR using topographic and morphologic codes. All tumours registered as benign in NCR were excluded from the analysis. Childhood cancer was defined as a cancer diagnosis before the age of 20 years.

In cases of multiple cancers, only the first malignant event was included in the analyses. When referring to endocrine tumours, malignancies of the thyroid, thymus, adrenal glands, parathyroid glands, pituitary gland, pineal gland, carotid body (glomus caroticum), and paraganglia were included.

### 2.3 Statistical Analysis

The association between MFS and cancer risk was assessed using Cox proportional‐hazard models expressed as hazard ratios (HRs) and corresponding 95% confidence intervals (CIs). Attained age was used as the underlying time scale, and all analyses were inherently adjusted for sex, birth year and birth county. Analyses examined the association between MFS and cancer risk at any age, and separately during adulthood (≥ 20 years) and childhood (< 20 years). For analyses of childhood cancer, only individuals born between 1st of January 1960 and 31st of December 2017 were included. These analyses were further adjusted for maternal and paternal age at birth (categorized as < 30, 30–39, > 39 years) and the highest parental education level of the mother and father (categorized as primary, secondary, postsecondary). Further analyses assessed the risk of solid tumours at specific ages (< 20, 20–39, 40–59, and ≥ 60 years) and the risk of site‐specific cancer during adulthood. Results were reported only when at least five cancer cases occurred among individuals with MFS. The proportional hazard assumption was tested using Schoenfeld's residuals. All data were prepared in SAS 9.4 and statistical analyses were conducted in Stata 16.

To reduce the risk of exposure misclassification, a sensitivity analysis was conducted using the method described above but restricted to individuals who had received a diagnosis of MFS on more than two occasions and/or had a confirmed disease‐causing variant in *FBN1*. Because the NPR was established in 1964 and reached nationwide coverage in 1987, a second sensitivity analysis was performed to assess cancer risk during a period with more complete data. In this analysis, individuals born in 1950 or later were included in the assessment of adult cancer, and individuals born 1970–2017 were included in the analysis of childhood cancer. Cancer outcomes were evaluated from 1970 onward, a period when most counties, including the largest ones, were already covered by the NPR.

In addition, a manual review was performed to further address potential exposure misclassification. This review included individuals with an elevated cancer risk and involved examining all registered ICD codes in the NPR and MBR for individuals with MFS and their relatives.

## 3 Results

### 3.1 Baseline Characteristics

We included 1544 individuals with MFS, born in Sweden between 1930 and 2017. More than 200 individuals had a genetically confirmed MFS diagnosis (*n* = 232), of whom 194 were born after 1960. Characteristics of the MFS cohort and the matched comparisons are presented in Table 1. The majority of individuals with MFS were male (57%). One differences between the MFS group and the matched comparisons were that advanced paternal age (> 39 years) was slightly more common in the MFS group (11.3% vs. 9.1%). Moreover, the proportion of parents with postsecondary education was lower in the MFS group (33.4% vs. 36.6%).

**TABLE 1:** Characteristics at baseline and end of follow‐up for all study participants.

	Marfan Syndrome	Matched comparisons
Total number of individuals	No (%)	No (%)
	1544	77,200
Sex		
Male	876 (56.7)	43,800 (56.7)
Female	668 (43.3)	33,400 (43.3)
Birth year		
1930–1949	208 (13.5)	10,400 (13.5)
1950–1969	341 (22.1)	17,050 (22.1)
1970–1989	497 (32.2)	24,850 (32.2)
1990–2009	443 (28.7)	22,150 (28.7)
2010–2017	55 (3.6)	2750 (3.6)
Maternal age		
< 30	898 (58.2)	46,675 (60.5)
30–39	531 (34.4)	26,614 (34.5)
> 39	39 (2.5)	1777 (2.3)
Missing data	76 (4.9)	2134 (2.8)
Paternal age		
< 30	613 (39.7)	32,349 (41.9)
30–39	639 (41.4)	33,556 (43.5)
> 39	175 (11.3)	7054 (9.1)
Missing data	117 (7.6)	4241 (5.5)
Highest parental education		
Primary	233 (15.1)	12,675 (16.4)
Secondary	531 (34.4)	29,206 (37.8)
Postsecondary	515 (33.4)	28,224 (36.6)
Missing data	265 (17.2)	7095 (9.2)
Reasons for end of follow‐up		
Cancer diagnosis	67 (4.3)	4429 (5.7)
Median age at diagnosis (SD)	51.0 (20.4)	59.0 (16.9)
Dec 31, 2017		
Median age at end follow‐up (SD)	31.6 (20.0)	35.1 (20.4)

### 3.2 Cancer Risk

Results for the association between MFS and cancer risk at different ages are shown in Table 2. In total, we observed 67 cancer cases among individuals with MFS, including 61 solid tumours. The overall risk of cancer at any age was not increased in individuals with MFS (HR 1.00, 95% CI 0.78–1.27), nor was the risk of solid tumours (HR 1.00, 95% CI 0.79–1.29).

**TABLE 2:** Risk of overall cancer, solid tumours, and specific malignancies in individuals with Marfan syndrome relative to comparisons, stratified by age, sex and cancer site.

Cancer risk		
	Cases of marfan/comparisons	HR (95% CI)
All cancer	67/4429	1.00 (0.78–1.27)
Age (years)		
< 20 (Born after 1960)	9/197	2.44 (1.25–4.80)^a^
≥ 20	58/4225	0.91 (0.71–1.19)
20–40	13/522	1.39 (0.80–2.42)
20–60	36/2118	1.06 (0.76–1.48)

Risk of solid tumours		
	Cases of marfan/comparisons	HR (95% CI)
All solid tumours	61/4003	1.00 (0.79–1.29)
Age (years)		
< 20 (Born after 1960)	7/126	2.97 (1.38–6.39)^a^
≥ 20	54/3873	0.93 (0.71–1.21)
20–39	11/455	1.34 (0.74–2.44)
40–59	23/1469	1.01 (0.67–1.53)
≥ 60	20/1949	0.73 (0.45–1.14)

Site‐specific cancer risk in adults		
	Cases of marfan/comparisons	HR (95% CI)
Breast	11/703	0.93 (0.51–1.70)
Prostate	8/652	0.94 (0.47–1.91)
Digestive organs	6/585	0.73 (0.33–1.64)
Female genitalia	5/265	1.16 (0.48–2.82)
Endocrine	8/176	2.86 (1.40–5.85)

^a^ Adjusted for maternal and paternal age and highest achieved level of education.

Among adults with MFS, 54 cases of solid tumours and fewer than five haematological malignancies/lymphomas were identified. The overall cancer risk was not increased when stratified by age 20–40 years (HR 1.39, 95% CI 0.80–2.42) or 20–60 years (HR 1.06, 95% CI 0.76–1.48). The risk of solid tumours in adults did not differ from that in matched comparisons (HR 0.93, 95% CI 0.71–1.21). Site‐specific cancers in the MFS cohort included malignancies of the breast, prostate, lung, digestive organs, skin (melanoma), head and neck, kidneys, male and female genital organs, central nervous system, lymphoma, leukaemia and endocrine organs. The risk of endocrine tumours was nearly three times higher in adults with MFS (HR 2.86, 95% CI 1.40–5.85), while no significant increases were observed for other cancer sites (Table 2). The absolute risk of endocrine tumours was 0.52% in MFS as compared with 0.23% in the comparison group.

We observed nine cases of childhood cancer in the MFS cohort, of which seven were solid tumours. The risk of childhood cancer was more than twofold higher in MFS (HR 2.44, 95% CI 1.25–4.80) (Table 2), with an absolute risk of 0.76% in MFS as compared to 0.33% in the unexposed cohort. The incidence rate of childhood cancer was 44.6 per 100,000 person‐years (95% CI 23.2–85.7) in MFS and 18.8 (95% CI 16.3–21.6) per 100,000 person‐years in the unexposed cohort, corresponding to an incidence rate ratio of 2.38 (95% CI 1.22–4.63).

When analysing solid tumours in childhood, the risk was nearly three times higher than in the comparison group (HR 2.97, 95% CI 1.38–6.39). The association between MFS and site‐specific childhood cancers could not be evaluated due to the small number of cases; however, cases of hepatocellular, testicular, and ovarian tumours, as well as tumours in the central nervous system, were observed in children with MFS.

### 3.3 Sensitivity Analysis

In the first sensitivity analysis, 946 individuals with MFS were included, of whom 36 had a registered diagnosis of cancer (see Table 3). The risk estimates were similar to the ones observed in the main analysis, showing no overall increased risk of cancer (HR 0.75, 95% CI 0.54–1.04) but an elevated risk of endocrine tumours. The risk of childhood cancer in individuals with MFS could not be assessed in this analysis, as the number of observed cases was too small (< 5).

**TABLE 3:** Sensitivity analysis; risk of cancer in individuals with the diagnosis of Marfan syndrome more than two times and/or a registered disease‐causing variant in *FBN1* relative to comparisons.

Cancer risk		
	Cases of marfan/comparisons	HR (95% CI)
	946/47300	
All cancer	36/2656	0.75 (0.54–1.04)
All solid tumours	32/2395	0.74 (0.52–1.05)
Endocrine tumours	6/105	3.23 (1.41–7.39)

The second sensitivity analysis included 1336 individuals with MFS, among whom 39 cancer cases were identified (see Table 4). In this analysis, the increased risk of endocrine cancer in adults was consistent with the main analysis. There were eight cases of childhood cancer, and the risk of childhood cancer was elevated for both overall cancer and solid tumours, consistent with findings of the main analysis.

**TABLE 4:** Sensitivity analysis; individuals with Marfan syndrome, including adults born 1950 and onwards, and children born 1970–2017. Risk of overall cancer and solid tumours in individuals with Marfan syndrome relative to comparisons, stratified by age, sex, and cancer site.

Cancer risk		
	Cases of marfan/comparisons 1336/66800	HR (95% CI)
Age (years)		
< 20 (Born after 1970)	8/183	2.27 (1.11–4.65)^a^
≥ 20 (Born after 1950)	31/1570	1.21 (0.85–1.73)

Risk of solid tumours		
	Cases of marfan/comparisons	HR (95% CI)
Age (years)		
< 20 (Born after 1970)	6/121	2.64 (1.16–6.01)^a^
≥ 20	29/1415	1.25 (0.86–1.81)

Site‐specific cancer risk in adults		
	Cases of marfan/comparisons	HR (95% CI)
Breast	6/286	1.18 (0.48–2.87)
Female genitalia	5/103	2.97 (1.20–7.35)
Endocrine	5/96	3.10 (1.25–7.66)

^a^ Adjusted for maternal and paternal age and highest achieved level of education.

In the manual review of individuals with MFS and an elevated cancer risk, a total of 17 individuals were included, comprising eight cases of endocrine tumours and nine cases of childhood cancer. All individuals with endocrine tumours had at least one registered code supportive of an MFS diagnosis (e.g., aortic dissection I71.0 (ICD‐10), or lens dislocation H27.1 (ICD‐10)), or had a relative diagnosed with MFS. Among individuals with childhood cancer, the majority had either a documented *FBN1* variant or an ICD code supportive of an MFS diagnosis. None of the children had a relative formally diagnosed with MFS; however, two had relatives with diagnoses suggestive of MFS.

## 4 Discussion

In this population‐based matched cohort study of 1544 Swedish individuals with MFS, we did not confirm the previously reported overall increased cancer risk in adults with MFS. However, we observed a nearly threefold higher risk of endocrine tumours in adulthood among individuals with MFS compared to their matched controls. No increased risk was detected for other site‐specific cancers. Moreover, children with MFS had a more than twofold higher risk of developing cancer, driven by solid tumours.

These findings partly contradict results published by Hsu et al., who reported 93 cancer cases and found an increased risk of tumours in adults with MFS at several sites, including the head and neck, oesophagus, stomach, colon and rectum, liver, female genital organs, prostate, bladder, ureter, kidney, and hematopoietic malignancies. In our study, the risk for endocrine tumours in individuals with MFS was nearly three times elevated, whereas Hsu et al. reported an odds ratio of approximately 10.5 for thyroid malignancies in MFS.

Multiple endocrine neoplasia type 2 (MEN2B) is a genetic syndrome caused by pathogenic variants in the proto‐oncogene RET (REarranged during Transfection). MEN2B is characterized by a marfanoid habitus and an increased risk of endocrine tumours, including medullary thyroid carcinoma, pheochromocytoma, and diffuse intestinal ganglioneuromatosis [30, 31]. Because of the phenotypic overlap, individuals with MEN2B may occasionally be misdiagnosed as having MFS. In our cohort, however, fewer than five endocrine tumours were located in the thyroid gland, and none were identified as medullary carcinoma, pheochromocytoma, or diffuse intestinal ganglioneuromatosis. Furthermore, five of the eight individuals with endocrine tumours and a diagnosis of MFS had ICD codes consistent with characteristic features of MFS. The remaining three had a relative with a confirmed MFS diagnosis, supporting the diagnostic validity. The increased risk of endocrine tumours persisted in both sensitivity analyses. Although some degree of diagnosis misclassification cannot be entirely excluded, our findings support a robust association between MFS and endocrine tumours, which we do not believe is due to phenotypic misdiagnosis of MFS in individuals with MEN2B.

The findings regarding endocrine tumours should, however, be interpreted in light of potential surveillance bias. Given the high prevalence of cardiovascular complications in MFS, transthoracic echocardiography is recommended for all individuals. In addition, from 18 years of age, vascular imaging of the thorax and abdomen using computed tomography or magnetic resonance imaging is often performed every 2–5 years [32]. This frequent imaging may lead to surveillance bias for tumours located in the thoracic and abdominal region, such as thymomas and adrenal tumours. Moreover, repeating imaging increases exposure to ionizing radiation, potentially contributing to a higher cancer risk. Further studies are needed to investigate the accuracy and the cause of the elevated risk of endocrine tumours observed in our study, as well as in the study by Hsu et al.

The individuals with MFS in this study were relatively young at the end of follow‐up, with a median age of 31.6 years, compared to 35.1 years in the comparison cohort. The median age at cancer diagnosis was also lower in the MFS cohort (51.0 compared to 59.0 years). These results support the findings of Hsu et al., who reported that adults with MFS develop malignancies at younger ages than adults without MFS [23].

The *FBN1* gene was identified as the causative gene for MFS in 1991 [2, 33]. Consequently, genetically confirmed MFS cases are more common among younger individuals, whereas the risk of misdiagnosis may be higher among older individuals. In the first sensitivity analysis, we included individuals who had received an MFS diagnosis on more than two occasions and/or carried a pathogenic *FBN1* variant to reduce misclassification. This analysis confirmed the absence of an increased overall cancer risk. Longer follow‐up studies are required to assess cancer risk in older individuals with MFS.

Germline genetic aberrations account for approximately 10%–15% of childhood cancer cases [34, 35, 36]. Certain neoplasms are strongly associated with childhood cancer predisposition (ChiCaP) syndromes, including optic nerve glioma, retinoblastoma, and kidney cancer [37]. Other childhood cancers such as leukaemia, hepatocellular carcinoma, Hodgkin lymphomas, germ cell tumours, astrocytoma, and glioblastomas have also been linked to ChiCaP [36, 37, 38]. In our MFS cohort, several of these tumours were present; however, site‐specific analyses could not be performed as there were too few cases.

Maya‐González et al. conducted a literature review suggesting an overrepresentation of childhood cancer in MFS and described two unrelated children with MFS and neuroblastoma [20]. No individual with neuroblastoma was identified in our cohort. However, our findings support an association between MFS and childhood cancer, with an increased risk observed for both overall cancer risk and the risk of solid tumours specifically, which remained consistent in the second sensitivity analysis. The elevated cancer risk in children was further supported by a significantly higher incidence rate and incidence rate ratio. However, the absolute risk of childhood cancer was still low (0.76% compared to 0.33%), and further studies are needed to investigate the role of MFS as ChiCaP syndrome.

## 5 Strengths and Limitations

To our knowledge, this population‐based study is the largest study to assess cancer risk in MFS and the first to specifically examine the risk of childhood cancer. The register‐based design enables long‐term follow‐up, with minimal selection bias, inclusion of a large proportion of individuals with MFS, and analyses by age group and cancer type.

The study design entails a risk of exposure misclassification, given the limited access to original medical records and the lack of opportunity for clinical re‐evaluation. However, we consider this risk to be low in the current study, as the diagnosis of MFS among individuals with either endocrine tumours or childhood cancer was well supported in the vast majority of cases based on manual review, including typical clinical features, corroborating genetic findings, or a consistent family history.

Another limitation of the study is that the NPR lacked full inpatient coverage before 1987, and outpatient data were unavailable prior to 2001. Although coverage increased gradually, with 18 of 26 counties included by 1973, the identification of MFS cases during the earlier years is likely incomplete. Furthermore, as the SCR was established in 1958, no earlier cancer diagnoses could be identified. To reduce the risk of missing childhood cancer cases during the first years of the study period, only individuals born after 1960 were included in the childhood cancer analyses. To further address this concern, a sensitivity analysis was performed including only children born from 1970 onward. The Swedish ICD‐9 code for MFS (759 W, Other specified malformations) was used from 1987 to 1996. The code was unspecific and included other malformation syndromes; consequently, we could not identify individuals with MFS from the NPR if they had only been diagnosed with an ICD‐9 code.

## 6 Conclusions

In this Swedish matched cohort study, we found a more than two‐fold increased risk of childhood cancer in MFS, driven by solid tumours. The overall cancer risk in adults with MFS was not increased, except for a nearly threefold increased risk of endocrine tumours. However, the absolute risk of both childhood and adult cancer remains low and does not motivate any altered screening recommendations. Further studies with longer follow‐up are warranted to evaluate the lifetime cancer risk in MFS.

## Author Contributions

**Ida Nordgren:** conceptualization, investigation, writing – original draft, methodology, validation, visualization, writing – review and editing, formal analysis, data curation, funding acquisition. **Anna Skarin Nordenvall:** conceptualization, investigation, methodology, validation, writing – review and editing, formal analysis, data curation, supervision. **Alexandra Wachtmeister:** methodology, investigation, writing – review and editing, formal analysis. **Fulya Taylan:** methodology, writing – review and editing. **Yunxia Lu:** methodology, writing – review and editing, formal analysis, supervision, investigation. **Christina Norrby:** methodology, investigation, writing – review and editing, formal analysis. **Anna Lindstrand:** supervision, methodology, writing – review and editing. **Giedre Grigelioniene:** conceptualization, methodology, software, data curation, supervision, resources, project administration, writing – review and editing, funding acquisition, investigation, visualization, formal analysis. **Giorgio Tettamanti:** conceptualization, funding acquisition, investigation, validation, methodology, writing – review and editing, project administration, supervision, data curation, visualization, resources, formal analysis.

## Funding

This work was supported by Cancer Research Funds of Radiumhemmet, 211293, Cancerfonden, 222057 PJ, Region Stockholm: 520136 ALF, Barncancerfonden, 24‐3504 Pj, PR2022‐0027, Vetenskapsrådet, 2021‐02860, 2022‐02647, 2022‐06312.379, Stiftelsen Promobilia, A24186, A25439, Stiftelsen Barnavård, 2025‐076, H.K.H. Kronprinsessan Lovisas Förening för Barnasjukvård, 2022‐00728, 2024‐043, and Karolina Widerströms Studie‐ och Stipendiefond.

## Ethics Statement

The study has approval from the Regional Ethical Review Board in Stockholm (Dnr 2018/1849–32).

## Conflicts of Interest

The authors declare no conflicts of interest.

## Data Availability

Pseudonymized personal data were obtained from the national Swedish Registry holders after ethics approval and secrecy assessment. Personal sensitive data can only be made available to researchers who fulfil legal requirements for access according to Swedish laws and regulations. For further information on data access, please contact Associate Professor Giorgio Tettamanti (giorgio.tettamanti@ki.se). Further data that support the findings of this study are available from the corresponding authors upon reasonable request.
